# Engineering the plant microbiome: synthetic community approaches to enhance crop protection

**DOI:** 10.3389/fpls.2025.1705289

**Published:** 2026-02-02

**Authors:** Ketankumar Panchal, Ankit Sudhir, Anil S. Prajapati

**Affiliations:** 1 Department of Biological Sciences, P. D. Patel Institute of Applied Sciences, Charotar University of Science and Technology, Anand, Gujarat, India; 2 Department of Biological Sciences, School of Sciences, GSFC University, Vadodara, Gujarat, India; 3 M. B. Patel Science College, Anand, Gujarat, India

**Keywords:** synthetic microbial communities, crop immunity, disease resilience, microbiome engineering, plant microbiome, rhizosphere microbiota, sustainable agriculture

## Abstract

The plant microbiome is essential for plant health; in particular, synthetic microbial communities (SynComs) offer a scalable, sustainable alternative to chemical pesticides. The concept has moved beyond single-strain inoculants, with SynComs being rationally designed using ecological principles, computational tools, and an understanding of how plants shape their microbial niche through root exudates and chemotaxis. Indeed, effective SynCom design requires a mechanistic understanding of microbe–microbe and host–microbe interactions. In real field settings, SynComs have been shown to suppress diseases in tomato, rice, wheat, and maize while enhancing yield. Inconsistent field performance, instability in formulation, regulatory challenges, and farmer adoption are among the pressing issues related to SynComs. In the foreseeable future, the integration of machine learning and gene-editing tools is expected to enable SynCom formulation with greater precision and impact. Favorable labor division and mutualistic relationships within a SynCom make it a more controlled and ecologically informed tool for modern agriculture.

## Introduction

**Figure 1:**
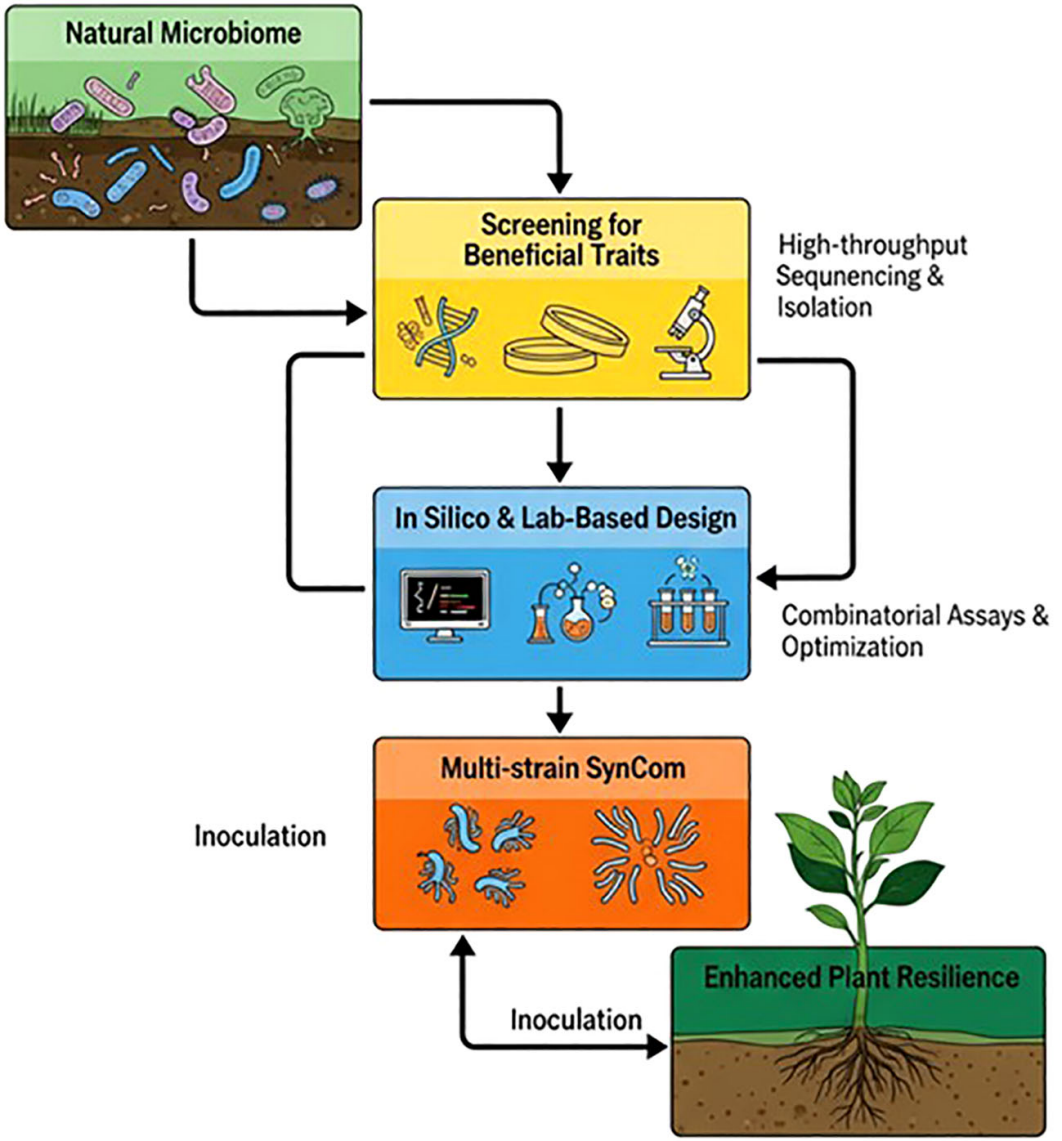
Workflow for SynCom design and implementation.

For decades, increasing global food demand has relied on agrochemicals and intensive farming practices. However, this approach has adverse environmental impacts, including soil degradation, groundwater contamination, resistance development in pathogens, and a loss of biodiversity (Gade et al., 2023; Zhang et al., 2023; Preethadevi et al., 2025). This has shifted the focus of agricultural research toward more sustainable, ecofriendly, biologically based strategies derived from the plant microbiome. Naturally, a diverse and complex array of microbes, including bacteria, fungi, and archaea, resides on plant surfaces and within tissues, providing essential roles in nutrient uptake, growth, stress tolerance, and immunity against pathogens (Trivedi et al., 2021; Preethadevi et al., 2025). Beneficial microbes protect plants by producing antimicrobial compounds as well as by activating the plant immune system (Berg et al., 2020; Pascale et al., 2020). However, environmental changes, host genetics, and agricultural practices can disrupt the balance of these microbial communities, leading to the loss of beneficial and consistent protective functions on a large scale and making plants vulnerable to disease (Porter and Sachs, 2020; Jing et al., 2024; Preethadevi et al., 2025). Among biologically based strategies, the use of microbial inoculants has gained considerable attention as an alternative to chemical pesticides and fertilizers. However, field application of traditional single- strain microbial inoculants often yields inconsistent results due to the complexity of microbial interactions, including adaptability to the soil environment, competition with native microorganisms, and lack of multifunctionality (Sammauria et al., 2020; Tariq et al., 2025). These limitations have encouraged researchers to use synthetic microbial communities (SynComs). A SynCom is a simple yet functional microbial consortium of selected microorganisms designed to promote more positive and predictable plant–microbe interactions that support plant growth and resistance (**Figure 1**) (Flores-Núñez et al., 2023; Jing et al., 2024; Tariq et al., 2025). Compared with single-strain inoculants, the SynCom approach offers a powerful platform to study microbe–microbe and plant–microbe interactions in a controlled environment before moving to the field, paving the way for next-generation bioinoculants (Jing et al., 2024). The use of SynComs provides multifaceted benefits, including pathogen suppression, stress tolerance, nutrient uptake, and immune modulation, for crop protection (Marin et al., 2021).

In SynComs, not one strain but many strains work together, providing functional redundancy as well as cross-feeding to suppress plant disease while behaving more stably and more reliably (Bai et al., 2015; Hassani et al., 2018). We now understand that a plant is not just a passive host; rather, it is active, and through plant root exudates and immune signaling, plants shape their own microbiome (Lebeis et al., 2015). Even better, the plant itself can be modified through genetic or physiological means to recruit specific beneficial microbes. Such modifications have the potential to turn the host into a recruitment platform for SynComs, as desired (Hu et al., 2018; Tkacz et al., 2018). Furthermore, SynComs can integrate cross-kingdom consortia that partner fungi and bacteria, leveraging complementary mechanisms for effective biocontrol efficacy and plant growth promotion (Minchev et al., 2021; Prigigallo et al., 2022; Kampa et al., 2025). This shift has helped move the focus from studying microbes in isolation toward understanding microbial interactions that shape plant health. However, significant gaps remain in understanding the factors that determine the success or failure of introduced SynComs under field conditions, because their establishment, persistence, and ability to provide effective plant protection are influenced by soil texture, host genotype, and existing microbiome (Shayanthan et al., 2022; Trivedi et al., 2022a). Additionally, formulation instability, inefficient delivery, unclear regulatory frameworks, and a lack of farmer trust limit real-world application (Busby et al., 2017; Ke et al., 2021; Beattie et al., 2024; Hanif et al., 2024). This review discusses how microbiome engineering can support more sustainable and ecofriendly farming systems by addressing design, testing, and deployment, with a focus on crop disease resilience supported by a real-world field case. It offers a roadmap for researchers, breeders, and policymakers to translate microbiome engineering from laboratory concept into a scalable and sustainable solution.

## Unpredictable nature of the natural plant microbiome and protection

**Figure 2:**
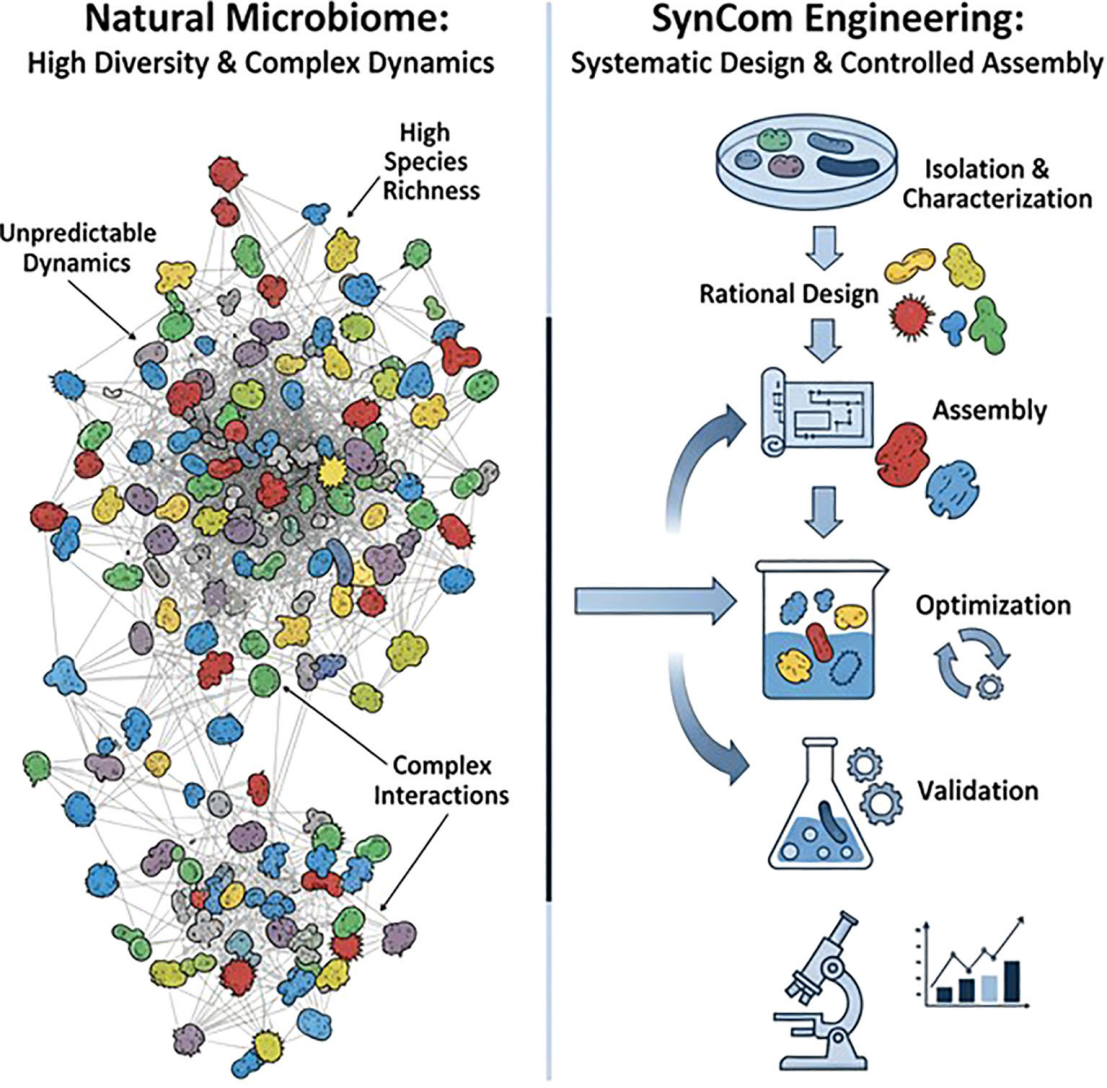
Comparison of natural microbiomes and SynCom engineering.

The unpredictable nature of the natural plant microbiome poses significant challenges in agriculture, especially for consistent crop protection (Sawant et al., 2025). Since the natural plant microbiome is closely associated with plant health, its inherent complexity and instability make it difficult to reliably harness beneficial traits for agricultural applications (**Figure 2**) (Trivedi et al., 2022a; Poupin and Gonzalez, 2024a; Poupin and Gonzalez, 2024b). One of the primary reasons for this unpredictability lies in the intricate interactions between the microbiome and the host plant (Chen et al., 2025). The unpredictability of the composition and function of a plant’s native microbial community results from multiple variables, including soil pH, moisture, nutrient availability, and soil texture, which shape microbial communities colonizing plants (Trivedi et al., 2022a; Jing et al., 2024; Poupin and Gonzalez, 2024b). Additionally, plant genetics and age play a significant role in shaping its microbiome; so even genetically identical plants can have different communities over their life cycle (Xiong et al., 2021; Zhang et al., 2023a). Even two adjacent plants can have microenvironmental differences that lead to differential microbial profiles in a single field, causing inconsistent protective function (Trivedi et al., 2022a; Park et al., 2023; Poupin and Gonzalez, 2024b). Moreover, pathogen invasion itself can disrupt host control of the rhizosphere, increasing compositional variability and creating an opportunity for colonization (Kuang et al., 2023). Environmental stochasticity, such as drought, temperature fluctuations, heat stress, and humidity, amplifies this variation and can alter the delicate balance of the microbial community, thereby favoring opportunistic pathogens over beneficial microbes (Trivedi et al., 2022b). For example, an antifungal strain might fail to grow under water-stressed conditions, limiting the plant defense system when it is most needed (Kuang et al., 2023; Ahmad et al., 2024). Reliance on this highly complex, environmentally sensitive, and dynamic communities makes it difficult to provide protection at larger scales. This unpredictability does not imply that natural systems are flawed but highlights the need for a more rational and controlled approach that can deliver consistent crop protection, creating an opportunity for the engineering of synthetic microbial communities.

The plant immune system does not act alone but is also shaped by the microbes that live around different plant parts. This “extended immune system” includes bacteria, fungi, and other organisms that can protect plants against pathogens (Berg et al., 2020). The balance of these microbial communities often determines whether a plant remains healthy or becomes diseased (Wei and Jousset, 2017; Carrión et al., 2019). Induced systemic resistance (ISR) is one way in which beneficial microbes “train” the plant immune system to respond faster and more strongly to pathogen attacks. Many root-associated bacteria, such as *Pseudomonas fluorescens* and *Bacillus subtilis*, are reported to trigger ISR by producing molecules such as siderophores, lipopeptides, and volatile compounds (Pieterse et al., 2014; Stringlis et al., 2018). ISR differs from systemic acquired resistance (SAR) in that it does not rely on direct pathogen infection but rather on beneficial partners (Kamle et al., 2020; Yu et al., 2022). ISR is triggered by beneficial microbes, including rhizobacteria (*Bacillus* spp., *Pseudomonas* spp.) and fungi (*Trichoderma* spp., mycorrhizae), through jasmonic acid/ethylene (JA/ET)-dependent pathways, whereas SAR operates through salicylic acid (SA)-dependent signaling. Beyond ISR priming, the microbiome directly suppresses pathogens through antibiosis and competitive exclusion for nutrients and colonization sites (Berendsen et al., 2012; Yu et al., 2022). The microbiome also protects crops by directly suppressing pathogens by producing not only antibiotics or enzymes that kill pathogens but also competing for nutrients and root space (Mendes et al., 2011; Berendsen et al., 2012; Yu et al., 2022). Fungal endophytes, such as *Trichoderma*, are known to provide crop protection against soil-borne pathogens through both competition and induction of ISR (Tyskiewicz et al., 2022; Guzman-Guzman et al., 2023). Healthy plants often harbor unique microbial signatures, known as pathogen-suppressive microbiomes, that support disease suppression (Schlaeppi and Bulgarelli, 2015; Xiong et al., 2021). Recent findings further emphasize that cross-kingdom interactions like *Pseudomonas*, *Bacillus*, *Trichoderma*, and mycorrhizal species can enhance plant immunity more effectively by activating both JA/ET and SA defence pathways. This provides broader-spectrum protection than single inoculants and suggests that synthetic consortia may mimic natural cooperation (Pieterse et al., 2014). Additionally, plant genotype (including cultivars) and environmental factors (soil type, nutrients, and climate conditions) also influence how specific microbes are recruited into the microbiome, contributing to plant immunity (Wagner et al., 2016; Hassani et al., 2018; Walters et al., 2018; Compant et al., 2019). Understanding these determinants is therefore critical before designing or applying SynComs for predictable crop protection.

## Design of synthetic microbial communities and their interactions

Development of SynComs with specific and desired functions is a precise approach to harness microbial benefits for plants, without attempting to replicate the complexity of natural ecosystems (Hacquard et al., 2017). The formulation process involves a systematic workflow, beginning with the selection of individual strains and continuing through their strategic assembly and validation, aiming to create stable and effective consortia (Roell et al., 2019; Berg et al., 2020; Mehlferber et al., 2024) (**Figure 2**). SynCom design follows either a bottom–up or top–down approach, each with unique advantages and limitations. The bottom–up approach (rational design) uses individually isolated microbial strains from plant rhizosphere and endosphere based on their beneficial criteria such as nutrient acquisition, pathogen suppression, and stress tolerance (Causevic et al., 2022; Wang et al., 2023; Clagnan et al., 2024; Lyu et al., 2024; Gómez-Lama Cabanás and Mercado-Blanco, 2025). This method offers the advantage of precise control over microbial composition, facilitating the testing of ecological principles such as competition and facilitation (Gómez-Lama Cabanás and Mercado-Blanco, 2025). However, the ecological stability and competitive fitness of these communities are often poor when introduced into native soil environments (Shayanthan et al., 2022). In contrast, the top–down approach simplifies the natural microbiome, systematically reducing its complexity through dilution or selective pressures while preserving core functional traits (Bernstein, 2019; Jing et al., 2024). This approach is advantageous because it helps the resulting SynCom to retain the natural resilience of the original community (De Souza et al., 2020). However, it may also introduce negative intermicrobial interactions, making community behavior less predictable. Recent studies suggest that the natural microbiome (NatComs) can also be optimized directly rather than replaced, providing a middle ground between uncontrolled natural complexity and a fully synthetic community (Causevic et al., 2022). These NatComs retain adaptive ecological coherence and are viewed as transitional stages in SynCom development. NatComs are derived from isolating and propagating intact microbial assemblies from the soil rhizosphere, preserving their functional interaction and network structure (Clagnan et al., 2024). The holobiont framework—viewing the plant and its associated microbiota as a single co-evolved ecological entity—provides theoretical justification for SynCom design strategies (Darriaut et al., 2025). A list of organisms used for the design of SynComs using either approach, along with their associated functional traits, is provided in **Table 1**. Researchers are increasingly employing hybrid strategies to leverage the strengths of both methods, combining the targeted selection of key microbial taxa from a bottom–up framework with the ecological resilience provided by a top–down approach. A promising middle ground involves using NatCom-derived microbial interactions in combination with targeted SynCom engineering, leveraging ecological realism while maintaining functional specificity (Mehlferber et al., 2024). This approach is supported by computational models and omics technologies that predict how different microbes will interact before their physical assembly (Northen et al., 2024a; Tariq et al., 2025). Root exudate chemistry and immune signals act as filters, enriching microbial partners with compatible functions. Therefore, SynCom design must integrate knowledge of host–microbe compatibility, emphasizing strains consistently present across genotypes and environments, often referred to as the core microbiome (Banerjee et al., 2018). These taxa form the core microbiome that stabilizes microbial assemblies under environmental perturbations. Core taxa frequently act as “hub” species, stabilizing community networks and enabling predictable functional outcomes (Banerjee et al., 2018; Wang et al., 2024; Rawstern et al., 2025). Co-occurrence networking, machine learning, comparative genomics, and functional screening are potential strategies for identifying hub taxa in SynCom design (Amit and Bashan, 2023; Wang et al., 2024). Integrating these hub taxa, including both bacterial and fungal members, can provide cross-kingdom stability and enhanced plant protection, validating the hub-centric design approach.

**Table 1:** List of microbial strains used for SynCom design by bottom–up and top–down approaches.

Microbe(s)	Functional trait(s)	Host/Environment	Design approach	Reference
*Bacillus*, *Pseudomonas*, and *Streptomyces*	Production of organic acids, phytohormones, and nutrient uptake	Wheat	Bottom–up	(Mažylytė et al., 2024)
*Serratia nematodiphila* EDR2, *Klebsiella variicola* EChLG19, *Bacillus thuringiensis* EML22, *Pantoea agglomerans* EMH25, *Bacillus thuringiensis* EBG39, *Serratia marcescens* EPLG52, and *Bacillus tropicus* EPP72	Nutrient cycling, biocontrol	Maize	Bottom–up	(Hernández-García et al., 2025)
Roteobacteria, Bacteroidota, Acidobacteriota, Actinobacteriota, and Firmicutes	Resilience under drought stress	*Brachypodium distachyon*	Top–down	(Yadav et al., 2025)
*Pseudomonas* sp., *Rhizobium* sp., *Ensifer* sp., *Microbacterium* sp., *Agromyces* sp., and *Chryseobacterium* sp.	Nutrient mobilization, phytostimulation, and biocontrol	Tomato	Bottom–up	(Montoya et al., 2025)
*Rhizobium*, *Ensifer fredii*, and *Rhizophagus intraradices*	Nutrient acquisition and stress response	Soybean	Bottom–up	(Wen et al., 2025)
*Bacillus* sp., *Acinebacter* sp., *Enterobacter* sp., *Xanthomonas* sp., and *Burkholderia* sp.	Plant growth promotion and alter the soil microbial community	Wheat	Bottom–up	(Liu et al., 2022)
*Achromobacter xylosoxidans* Z2K8, *Burkholderia* sp. Z1AL11, *Klebsiella variicola* R3J3HD7, *Kosakonia pseudosacchari* Z2WD1, *Pantoea ananatis* E2HD8, *Pantoea* sp. E2AD2, *Phytobacter diazotrophicus* Z2WL1, *Pseudomonas protegens* E1BL2, and *P. protegens* E2HL9	Biocontrol and ISR stimulation	Maize	Bottom–up	(De la Vega-Camarillo et al., 2023)
*Variovorax paradoxus*, *Novosphingobium subterraneum*, *Hydrogenophaga pseudoflava*, and *Acidovorax* sp.	Nutrient acquisition and improving crop production	Rice	Bottom–up	(Ma et al., 2024)

The design of SynComs has evolved beyond simple co-inoculation to data-driven omics approaches such as genomics, transcriptomics, metabolomics, and proteomics (Kamath et al., 2025). These approaches provide integrated molecular-level insights into microbial diversity, functional genes, and regulatory pathways within the rhizosphere (Kamath et al., 2025). This information guides the design of functional outcomes by selecting microbial strains with specific beneficial properties, such as nutrient solubilization, phytohormone production, and pathogen suppression (Singh et al., 2025). Understanding pathway partitioning and well-defined microbial interactions can mitigate limitations observed with monocultures, such as metabolic burden and labor division (Northen et al., 2024a). Moreover, optimization of SynComs design includes enhancing resource utilization, replacing less effective members with functional equivalents, improving microbial communication and spatial relationship within the community, which favors a more robust SynComs design for deployment in diverse agricultural settings (Northen et al., 2024a).

## SynCom-mediated crop protection

SynComs provide a promising strategy to strengthen plant immunity by combining the functional diversity of multiple microbes (Hacquard et al., 2017). Unlike single-strain inoculants, SynComs can activate plant defenses through different molecular mechanisms at the same time, making the response stronger and more reliable (Zhang et al., 2019; Zhang et al., 2023a). Plant protection by SynComs involves both direct and indirect mechanisms. Direct mechanism involves pathogens suppression by either antibiosis or competition. In antibiosis, antimicrobial compounds released by SynCom directly kill or inhibit the growth of pathogens (Liu et al., 2017; Liu et al., 2022). The production of lipo-peptides by *Bacillus* species disrupts the cell membranes of pathogenic fungi and bacteria (Rossmann et al., 2020; Sreedharan et al., 2023). In competitive exclusion, SynCom microbes compete with pathogens for essential resources, and colonization sites lead to competitive exclusion, avoiding their colonization (Wang et al., 2023; Wang and Sugiyama, 2024; Wang et al., 2024). Production of siderophores sequesters nutrients like iron, effectively competing with pathogens for nutrient availability (Berendsen et al., 2012; Liu et al., 2017; Liu et al., 2022). Indirect protection is mediated through host-microbe communication, which includes ISR, signaling, and communication, which is mainly controlled by the jasmonic acid (JA) and ethylene (ET) pathways (Pieterse et al., 2014; Yu et al., 2022). Induction of the plant immune system by SynCom leads to a broad-spectrum, long-lasting defence against a wide range of pathogens and threats (Vannier et al., 2019; Niu et al., 2020). When beneficial microbes are recognized by the plant immune receptors, the activated immune system enables the host to mount a faster and more potent defence response upon subsequent pathogen attack (Van Wees et al., 2008). In crops such as maize and tomato, SynComs application has been reported to activate JA/ET-responsive genes for rapid response of defense-related compounds such as callose and stronger resistance to pathogens (Niu et al., 2017; Carrión et al., 2019; Niu et al., 2020). Cross-kingdom SynComs combining fungal and bacterial partners can also exhibit synergistic immune activation. Modulation of root exudates is another important mechanism that controls the activity and recruitment of SynCom members. SynComs can release bioactive molecules like siderophores, phenazines, lipopeptides, and volatile organic compounds (VOCs), which directly inhibit pathogens and spike plant immune signaling in neighbouring plants, establishing a collective protective effect (Harman et al., 2021; Yang et al., 2023; Gómez-Lama Cabanás and Mercado-Blanco, 2025). In *Arabidopsis*, MYB72-dependent secretion of coumarins selectively enriches root bacteria that promote ISR and restrict pathogen growth (Stringlis et al., 2018). Dynamic, two-sided dialogue between SynComs and the plant, as molecular crosstalk, involves microbial quorum-sensing signals that interact with plant transcriptional networks, and plant exudates that modulate microbial gene expression in return (Tian et al., 2025). Such coregulated signaling ensures mutual adaptation and functional stability under field conditions. When SynCom-compatible host genotypes are matched with appropriate microbial consortia, the resulting plant–microbe holobiont exhibits enhanced resilience compared to either strategy alone (Trivedi et al., 2020a).

Recently, SnyComs have demonstrated promising capabilities for crop protection against various biotic and abiotic stresses. Carefully designed SynComs have been shown to effectively suppress the fungal pathogen *Rhizoctonia solani AG8* in wheat (Northen et al., 2024a). This suppression is achieved through the induction of both induced systemic resistance and systemic acquired resistance, as well as the production of antimicrobial compounds and siderophores. Enhancement of soil functional microbial abundance and multifunctionality has been shown to protect strawberries from soil-borne diseases (Ali et al., 2026). Beyond protection from biotic stressors, SynComs are also being engineered to enhance crop tolerance to abiotic stressors such as drought, salinity, and heavy metal toxicity (Dubey et al., 2025; Tripathi et al., 2025). Thus, with the help of SynComs, plants can restructure their microbiomes to cope with diverse stressors.

## SynComs vs. isolated single PGPR: the case for a systems-level approach

For decades, the agricultural practice has relied on the application of single, isolated strains of Plant Growth-Promoting Rhizobacteria (PGPRs) (Akinrinlola et al., 2018). Despite demonstrating remarkable capabilities under controlled laboratory conditions, their application in agricultural fields has yielded inconsistent and often disappointing results (Kaminsky et al., 2019; Basu et al., 2021; O’Callaghan et al., 2022). The disparity between simplified laboratory settings and the complex, competitive, and dynamic nature of soil is the fundamental reason for failure in most cases. When a single strain is introduced into a field, it faces difficulty establishing a stable population while simultaneously competing for limited resources and physical space against an already adapted and genetically diverse resident microbial community. Competition and the lack of synergistic interactions with other microbes lead to a rapid decline in the inoculated population, rendering the treatment ineffective before the end of the crop season (Kaminsky et al., 2019; Trivedi et al., 2020b; O’Callaghan et al., 2022; Trivedi et al., 2022b). Furthermore, PGPR lacks functional diversity to respond to multiple stressors, making it vulnerable in dynamic environments. The reliance on a “one-bug, one-function” paradigm is a key reason for the inconsistent performance of single inoculants across different soil types, climates, and host–plant genotypes (O’Callaghan, 2016; Basu et al., 2021; O’Callaghan et al., 2022). These inherent limitations can be overcome by the use of SynComs, leveraging principles of microbial ecology and network stability (Jing et al., 2024). The design criteria for SynComs depend on the intended functional outcomes rather than persistence alone (Delgado-Baquerizo et al., 2025). For applications requiring continuous biocontrol, long-term colonization, nutrient mobility, plant protection, and native microbiome modulation, SynCom members should exhibit stable root colonization, biofilm formation, and competitive fitness against native microbiota. Conversely, for applications focused on immune priming and transient stress protection, persistence is less critical; instead, the consortium should rapidly activate plant defense pathways (Yu et al., 2022). A SynCom directly leverages the power of community-level interactions. The collective effect of combined compatible strains with synergistic interactions can produce a far greater effect than the individual strains (Cheng et al., 2024). Therefore, effective SynCom design requires matching persistence strategies to functional objectives. The functional redundancy of this multistrain approach ensures that if one strain is outcompeted or becomes dormant, another can compensate, acting as a “safety net” and providing stability that is impossible for a single-strain inoculant (Wang et al., 2023; Wang and Sugiyama, 2024; Wang et al., 2024; Wang et al., 2025). However, this ecological advantage comes with formulation challenges, such as maintaining compatibility, metabolic balance, and scalability, which must be optimized before field application (Jing et al., 2024; Duran et al., 2025). A well-designed SynCom can provide broad-spectrum, multifunctional protection against multiple biotic and abiotic stresses simultaneously, offering a comprehensive and sustainable solution that a single microbe cannot offer (Jing et al., 2024). The evolution from single- to multistrain biofertilizers and ultimately to SynComs represents a progressive understanding of microbial ecology in the soil rhizosphere and a step toward more complex yet effective biological solutions (Singh et al., 2025).

## Deployment of SynComs in agriculture

The careful design of SynComs can provide multifaceted benefits, including enhanced plant immunity, improved nutrient use efficiency, and increased stress tolerance within a single system (Vorholt et al., 2017; Trivedi et al., 2020b). In real field settings, researchers have demonstrated significant improvements in both disease resistance and crop yield. A study on the model plant *Arabidopsis thaliana* showed that a multistrain community could significantly reduce disease symptoms from the pathogen (Kudjordjie et al., 2023). A two-strain SynCom composed of *Pseudomonas fluorescens* and *Bacillus velezensis* was applied as a tomato seed coating and was found to reduce vascular wilt caused by *Fusarium oxysporum* in greenhouse trials. It maintained disease suppression across three field seasons and increased fruit yield (Chaturvedi et al., 2022). The induction of jasmonate-mediated systemic resistance by beneficial microbes is a key mechanism for blast control in rice. The foundational work by Someya et al (Someya et al., 2002). demonstrated that the root-associated bacterium *Serratia marcescens* alone could reduce lesion area by approximately 65% and increase grain yield (Someya et al., 2002). Building on this principle, subsequent research has explored the potential of combining such resistant-inducing bacteria with other biocontrol agents, such as *Streptomyces* spp., to form more robust SynComs (Law et al., 2017; Du et al., 2025). Application of SynComs to maize seeds reduced anthracnose stalk rot in greenhouse trials. When combined with a chitosan-based encapsulation matrix, efficiency was maintained in rainfed fields where chemical fungicides were ineffective due to wash-off (Sharma et al., 2024). Beyond direct pathogen suppression, SynComs also hold promise for mitigating mycotoxin contamination in grains, a critical food safety issue associated with pathogens such as *Fusarium graminearum* (Zhang et al., 2023b).

One of the most critical innovations has been the development of protective carrier systems that enhance microbial viability during storage and after soil application. For instance, alginate–chitosan microcapsules have been shown to extend the shelf life of *Pseudomonas fluorescens*-based SynComs to over 6 months at ambient temperature while maintaining 85% cell viability—a crucial advantage for distribution in tropical countries such as India, where cold-chain infrastructure is limited (Safari et al., 2020; Khan et al., 2023). While these examples demonstrate SynCom’s potential, field performance remains highly context-dependent, with success or failure determined by interacting environmental, biological, and methodological factors (Delgado-Baquerizo et al., 2025). Recent ecological frameworks emphasize that many microbiome engineering efforts fail not due to inherent limitations of SynComs but due to inadequate application of ecological principles during the design, colonization, and maintenance stages. SynCom success requires: (i) optimized diversity and abundance matched to functional goals; (ii) niche complementarity, in which SynCom members occupy distinct ecological roles, minimizing competitive exclusion; and (iii) host-associated niche engineering, where plant root exudates and immune signals are leveraged to create favorable recruitment environments for introduced consortia (Darriaut et al., 2025; Henry and Bergelson, 2025). Similarly, lignin-based hydrogels not only protect microbes from desiccation and UV stress but also slowly release nutrients that enhance root colonization compared to liquid inoculants (Hachimi Alaoui et al., 2023). Seed coating has emerged as the most scalable and farmer-friendly delivery method. Research on seed coatings with beneficial microbes, including “Trichoderma”, shows reduced fungicide leaching and maintained efficacy (Gubišová et al., 2021; Turkan et al., 2023; Gubišová et al., 2024), paving the way for commercial SynCom-coated seeds. However, delivery timing is equally critical. SynComs applied at sowing or during the early seedling stage consistently outperform those applied postinfection, as early root colonization is essential for establishing ecological niches before native microbes or pathogens dominate (Arnault et al., 2024; You et al., 2025). Region-adapted SynComs, tailored to local soil microbiota, consistently outperform generic formulations under diverse field conditions by improving compatibility, colonization, and crop outcomes. This strategy is key to ensuring reliable SynCom performance across heterogeneous agricultural landscapes (Jiang et al., 2023; Li et al., 2024).

Apart from soil type and host genotype, agricultural practices and environmental stressors also affect SynCom performance. SynComs applied to one crop may fail to establish in subsequent crop cycles due to changes in root exudate profiles and pH that disrupt the microbial niches. This limitation can be addressed by designing “rotation-resilient” consortia that include generalist taxa capable of colonizing multiple hosts (Duncker et al., 2021; Mousa et al., 2024). Similarly, many SynCom members lose metabolic activity under drought or salinity stress, but their formulation with osmotolerant *Bacillus* and EPS-producing *Pseudomonas* maintains protective function through cross-feeding and biofilm matrices (Prigigallo et al., 2022; Qiao et al., 2024; Zhao et al., 2024). SynComs derived from salt-tolerant environments can effectively confer salt stress resistance to vulnerable crops. By assembling 33 different SynComs from bacterial strains previously isolated from salt-acclimatized mung bean roots, researchers were able to alleviate salt stress, providing a practical solution for agriculture. This strategy extends beyond food crops. Another study found that a SynCom from the rhizosphere of the salt-tolerant Euphrates poplar (*Populus euphratica*) conferred salt resistance to a different poplar species (*Populus alba × Populus glandulosa*) (Li et al., 2025; Schmitz et al., 2022; Dubey et al., 2025). These findings highlight that SynComs must be stress-tested not in isolation but within the dynamic reality of farms.

In agriculture, the application of SynComs is expanding, as they are being explored for enhancing soil fertility, mitigating climate/environmental change, restoring damaged microenvironments, and aligning with principles of sustainable agriculture (Dubey et al., 2025). Furthermore, SynComs are being investigated for composting to accelerate lignocellulose degradation and humus formation (Chen et al., 2025), as well as for the phyllosphere to improve nutrient uptake, protect against pathogens, and enhance environmental resilience on plant leaves and stems (Sarver et al., 2025). Therefore, successful SynCom deployment requires moving beyond generic “one-size-fits-all” formulations toward context-adapted, ecologically informed designs validated under realistic field conditions, with transparent reporting of both successes and failures.

## Limitations and future directions

Despite the many advantages of engineering and validating SynComs, several notable challenges remain before this technology can be applied in agriculture. These limitations span biological, ecological, regulatory, and ethical dimensions. A primary challenge is ensuring that SynComs remain stable and effective under real-world field conditions, as their efficacy may be affected by soil type, climate, native microbial populations, and plant genotype (Köhl et al., 2019; Trivedi et al., 2020b; Shayanthan et al., 2022; Delgado-Baquerizo et al., 2025). The lack of standardized protocols for SynCom development contributes to the difficulty of comparing or replicating results across studies (Hassani et al., 2018; Cheng et al., 2024; Northen et al., 2024b). Critically, many microbiome engineering efforts fail due to inadequate ecological design principles rather than technical constraints. Common failure modes include: (i) loss of key functional microorganisms during colonization due to competitive exclusion by resident microbes; (ii) disruption of functional links between introduced strains and plant; (iii) niche overlap, where SynCom members compete for the same resources rather than exhibiting complementarity; and (iv) insufficient consideration of niche dynamics governing how microbes establish, persist, and function in complex soil environments (Darriaut et al., 2025; Henry and Bergelson, 2025). Compared to chemical fertilizers, the large-scale manufacturing of multistrain inoculants presents cost-effectiveness and scalability challenges (Trivedi et al., 2022b; Jing et al., 2024). A significant hurdle is the lack of standardized protocols, community standards, and benchmarks, which altogether hinder efficient research progress and SynCom deployment (Northen et al., 2024a). Furthermore, the lack of regulation governing the safe use of SynComs constrains their deployment and hinders innovation (Vorholt et al., 2017; Compant et al., 2019; Gupta and Pandey, 2020). Introducing SynComs into natural soils poses a risk of long-term ecological consequences (Köhl et al., 2019; Hao et al., 2024). Additionally, public concerns about the ecological impacts of introduced microbes have led to rejection at the farmer level despite proven efficacy (Wunderlich and Gatto, 2015; Hunt and Wald, 2020).

The engineering of SynComs is moving beyond traditional trial-and-error methods toward a rational, data-driven design process. This shift is powered by advances in multiomics technologies (genomics, transcriptomics, proteomics, metabolomics) and computational modeling, which together enable a holistic understanding of microbial interactions and functions within both natural and synthetic communities (Subramanian et al., 2020; Picard et al., 2021; Brombacher et al., 2022). The integration of artificial intelligence and machine learning can help in predicting functional interactions to design SynComs for specific soil and cropping conditions (Wang et al., 2024; Tariq et al., 2025). Additionally, with the help of CRISPR-Cas gene-editing technology, researchers can precisely modify beneficial microbes to enhance their functions (Arroyo-Olarte et al., 2021; Wei and Li, 2023). Along with microbial engineering, microbiome-assisted breeding selects crop varieties based on their ability to recruit beneficial microbial consortia (Compant et al., 2019; Marco et al., 2022). The integration of digital agriculture with soil sensors and drone-based imaging helps monitor and adapt microbial applications (Paul et al., 2022). Also, pushing for a harmonized regulatory framework that considers SynComs as ecological inoculants rather than pesticides can increase their usage multifold (De Souza et al., 2020; Delgado-Baquerizo et al., 2025). Advances in SynComs may create the need for a global SynCom repository that provides open-access data, allowing researchers to refer to previously described SynCom designs. Public engagement is required to change the perception of SynComs; microbiome literacy programs will improve farmers’ trust and adoption (Sullivan et al., 2024). In addition to the future directions mentioned, the broader applicability of SynComs to other horizons can be include (a) the establishment of community standards, protocols, and benchmarks (Northen et al., 2024a); (b) integration of AI and omics technologies (Kamath et al., 2025); (c) greater emphasis on enhancing the ecological fitness and stability of SynComs (Velte et al., 2025); (d) integration of arbuscular mycorrhizal fungi for persistent efficacy (Zeng et al., 2025); and (e) SynCom design for specific agricultural challenges (Tripathi et al., 2025). To achieve sustainable agriculture and global food security in the near future, SynComs are capable of becoming a central pillar when technological innovation and practical application are applied in combination.

## Conclusion

SynComs represent a paradigm shift from reductionist single-strain approaches toward ecologically informed, systems-level strategies for sustainable crop protection. Synthetic microbial communities provide a practical and scalable solution for this purpose. Realizing their full potential will require interdisciplinary integration of microbial ecology, computational design, host breeding, and adaptive delivery systems tailored to local agroecosystems. In disease-resilient agricultural practices, a predictable, robust, and cost-effective SynCom design will require additional interdisciplinary efforts. SynComs have the potential to become more than a tool—they can transform laboratory innovation into field reality.
